# Possible Risk of Thrombotic Events following Oxford-AstraZeneca COVID-19 Vaccination in Women Receiving Estrogen

**DOI:** 10.1155/2021/7702863

**Published:** 2021-10-25

**Authors:** Ava Soltani Hekmat, Kazem Javanmardi

**Affiliations:** Department of Physiology, Fasa University of Medical Sciences, Fasa, Iran

## Abstract

People who receive the ChAdOx1 nCoV-19 vaccine, particularly perimenopausal women who are on birth control or postmenopausal women who take estrogen supplements, may experience thrombosis and thrombocytopenia. Estrogen and the ChAdOx1 nCoV-19 vaccine both have the potential to cause thrombus in different ways. Some postmenopausal women who are also taking estrogens may develop thrombosis and thrombocytopenia after receiving the ChAdOx1 nCoV-19 vaccine. Therefore, women are encouraged to stop taking drugs containing estrogen before receiving this vaccine. Furthermore, consuming fish oil can help reduce the risk of developing blood clots among women who are in the luteal phase and, thus, have high estrogen levels. In addition, ChAdOx1 nCoV-19's side effects in young women could be mitigated by administering it during the follicular phase.

## 1. Thrombose and Thrombocytopenia Induced by Vaccination

The ChAdOx1 nCoV-19 (henceforth referred to as Oxford-AstraZeneca) vaccine is classified as a recombinant chimpanzee adenoviral vector, and various countries have used it to treat cases of severe acute respiratory syndrome coronavirus 2 (SARS-CoV-2, more commonly known as COVID-19) [1, 2]. A prothrombotic syndrome was found in a small percentage of people who received the Oxford-AstraZeneca vaccination. Subsequently, identical results were observed in a few people who received the Janssen COVID-19 vaccine (Ad26.COV2.S), which, like the Oxford-AstraZeneca vaccination, is based on an adenoviral vector.

This condition is known as vaccine-induced immune thrombotic thrombocytopenia (VITT) [1]. The prevalence of VITT is unclear; though, it seems to be very rare. Most studies have reported few cases of illness among tens of millions of vaccinated people [1]. The most significant incidence was reported in a study conducted in Norway. In this study, five VITT cases were identified from among about 130,000 people who had been vaccinated with Oxford-AstraZeneca. This translates to an incidence rate of 1 in 26,000 cases [2].

The Oxford-AstraZeneca vaccines work by taking advantage of adenoviruses that carry DNA that encodes the spike protein, which is linked to COVID-19. This DNA is subsequently used by cells to make the spike protein, enabling the body to develop an immune response against it [3].

Clots associated with AstraZeneca occur in unusual parts of the body, such as the abdomen or brain, and are associated with low platelet levels. These characteristics are also seen in a disorder known as heparin-induced thrombocytopenia (HIT). Current knowledge suggests that HIT is initiated when heparin binds to a protein known as platelet factor 4 (PF4). As part of the resultant immune response, antibodies that protect against PF4 are produced. Platelets are destroyed, and clot-promoting materials are released as a result. However, it is unknown what specific event triggers this response when heparin is absent [3].

However, in all cases reported to date, only first dose of the Oxford-AstraZeneca vaccine generated thrombocytopenia, thrombosis, a very high d-dimer level, and low or normal fibrinogen levels. Details about this VITT pathogenesis are still unknown. Further studies are needed to confirm whether pathologic platelet-activating anti-PF4 antibodies, unrelated to heparin therapy, and are linked to vaccination against SARS-CoV-2 [3]. These patients have antibodies that bind to PF4-polyanion complexes at high levels [4, 5]. Unlike heparin-induced thrombocytopenia, antibodies can bind to PF4 even when heparin is not present [5].

It is unclear which of the vaccine's protein components play a potential role in platelet activation. Electrostatic interactions between positively charged PF4 and negatively charged heparin promote the formation of the PF4-heparin complex in HIT. Because adenoviruses have a negatively charged surface, the PF4 produced from platelets could form an immunogenic combination with adenoviral particles. This complex can then bind to platelets, causing VITT [6].

VITT can also be caused by the direct interactions between an adenoviral vector and platelets. Some adenoviruses have been found to bind to platelets through the coxsackie and adenovirus receptor (CAR), which is the first stage of thrombocyte viral entrance. Also, in vivo research has shown that a high adenoviral load in the blood may cause acute thrombocytopenia [6]. The delivery of adenoviruses activates platelets and leads to platelet aggregation [7].

In certain people, another possible cause of VITT is inflammatory reactions. Clinical findings indicate that most patients exhibit abnormal proinflammatory symptoms beginning eight to 12 hours after immunization and lasting 12 to 24 hours [8].

Although it has been shown that anti-SARS-CoV-2 spike protein antibodies do not crossreact with PF4, crossreactivity could occur between antivector antibodies and PF4 [6].

Anti-PF4 antibodies stimulate endothelial cells, monocytes, and neutrophils in addition to platelets. The activation of these other cell types increases the risk of thrombosis [1].

VITT's risk factors are unclear. However, preliminary findings indicate that females and younger people are at increased risk [1].

According to European data, women under 55 are at an especially high risk for blood clotting after receiving this vaccine [9]. In one study conducted in Germany and Austria, 11 individuals experienced thrombosis or thrombocytopenia after receiving the Oxford-AstraZeneca vaccine. Nine of these 11 patients were women, and they had a median age of 36 years [1]. Platelet-activating antibodies that act against PF4 caused this VITT. In another study, four of five cases were female. These individuals experienced venous thrombosis and thrombocytopenia seven to 10 days after receiving their first dose of Oxford-AstraZeneca. Antibodies to platelet factor 4-polyanion complexes were elevated in all of these patients [2]. In yet another study, 70% of 23 patients with VITT were under 50 years old, and 14 individuals were female [5]. Most of the patients in these studies were women under the age of 50, some of whom were taking oral contraceptives or undergoing estrogen replacement therapy [4].

## 2. Female Hormones and Thrombosis

Estrogen may increase the risk of blood clotting among vaccine recipients. Estrogen is known to impact immune cells' responses to flu vaccines, and so it is reasonable to propose that it might have a similar impact for other vaccines, including Oxford-AstraZeneca. Women are not naturally at a higher risk than men for developing blood clots. However, pregnancy and the use of birth control pills have hormonal effects that increase the risk of blood clotting in women [10].

Blood clotting is a natural response to a vascular injury as the body attempts to prevent excessive bleeding. The first step in the blood clotting process is the transient adhesion of platelets, first to collagen bound von Willebrand factor and then to the collagen matrix. These bound platelets then produce an ADP-, serotonin-, von Willebrand factor-, and fibrinogen-containing secretion while also synthesizing thromboxane A2. This process causes circulating platelets to form blood clots as the fibrinogen present in platelets binds to *α*IIb*β*3 integrin [11].

Scientific reports on estrogen's effects on platelet activation are inconsistent [11, 12]. Nevertheless, gender differences have been observed in terms of platelet properties, perhaps due to differences in sex hormones [13, 14]. Furthermore, it is known that hormonal changes that occur throughout the menstrual cycle can cause variations in many platelet characteristics and functions [15–17]. For example, Tarantino et al. demonstrated periodic fluctuations in the adhesion of platelets to collagen that was correlated with plasmatic estrogen peak levels [18].

Research also indicates that platelets' affinity to fibrinogen varies throughout the menstrual cycle; specifically, it is higher during the luteal phase than the follicular phase [14]. Oral estrogen, either alone or in combination with a progestogen, increases the risk of venous thromboembolism, especially in the first year of use. This risk disappears four months after stopping treatment [19]. Also, researchers have observed that postmenopausal present has lower levels of activated GPIIb-IIIa and P-selectin than premenopausal women [20].

## 3. Menopause and AstraZeneca Vaccine

Menopause is a biological event in which ovarian aging causes the female body to stop menstruating and ovulating. Menopause also causes the body's levels of progesterone and estrogen to decrease. As a result, estrogen-induced endometrial hyperplasia and adenocarcinoma are risk factors for postmenopausal women, who sometimes undergo progestogen to estrogen therapy to reduce these risks [10].

A study on roughly 16,000 women carried out by the Women's Health Initiative explored how oral menopausal hormone therapy might create specific risks and benefits among postmenopausal women. The researchers treated postmenopausal women with conjugated estrogens and medroxyprogesterone acetate and found that this treatment increased the likelihood of developing thrombotic events, coronary disease, and breast cancer [21].

Women who consume oral contraceptives containing estrogen tend to exhibit increases in the concentrations of clotting factors II, VII, X, XII, factor VIII, fibrinogen, and thrombin activatable fibrinolysis inhibitor in the plasma [22]. However, these increases vary significantly from one factor to the next. For example, the most noticeable increase is associated with factor VII, whereas the lowest increase is associated with factor VIII [23]. Research shows that oral contraceptives with desogestrel as an ingredient generate a more significant increase in factor VII concentration (26-32%) than similar contraceptives containing levonorgestrel (9-12%) [22, 23].

Such a drastic increase promotes thrombus formation while hindering the body's ability to decompose clots. These effects are somewhat offset by the fact that these contraceptives slightly decrease the concentration of factor V, which initiates the transformation of prothrombin (II) into thrombin (IIa) [22, 23]. This mechanism might seem beneficial at first; however, the resultant reduction in thrombus risk is essentially negated considering that factor V (in combination with protein S) inhibits factor VIII [24, 25].

A previous study found that consuming fish oil—which can induce a hypocoagulant state—reduced the levels of fibrinogen, factor V, and thrombin in the plasma. Particularly, subjects with high fibrinogen carrying *α*-chain fibrinogen polymorphism exhibited reduced thrombin generation and fibrinogen clustering [26].

## 4. Conclusion

Postmenopausal women who received estrogen-containing drugs may be prone to developing thrombosis and thrombocytopenia after being inoculated with the Oxford-AstraZeneca vaccine. Therefore, this vaccine should be administered to such women with great caution. It is strongly recommended that women who take estrogen-containing oral contraceptives discontinue use before receiving the Oxford-AstraZeneca vaccine. These women are also encouraged to consume fish oil before being vaccinated, as this can reduce the risk of clots. To further reduce the risk of thrombosis and thrombocytopenia, women should plan to receive this vaccine during the follicular phase of the menstrual cycle.

## Data Availability

No datasets were generated or analyzed during the current study.
